# Neo-sex chromosomes in the black muntjac recapitulate incipient evolution of mammalian sex chromosomes

**DOI:** 10.1186/gb-2008-9-6-r98

**Published:** 2008-06-14

**Authors:** Qi Zhou, Jun Wang, Ling Huang, Wenhui Nie, Jinhuan Wang, Yan Liu, Xiangyi Zhao, Fengtang Yang, Wen Wang

**Affiliations:** 1 CAS-Max Planck Junior Research Group, State Key Laboratory of Genetic Resources and Evolution, Kunming Institute of Zoology, Chinese Academy of Sciences (CAS), 32# Jiao-chang Road, Kunming, Yunnan 650223, People's Republic of China; 2 Graduate School of Chinese Academy Sciences, 19# Yu-quan Road, Beijing 100039, People's Republic of China; 3 The Institute of Human Genetics, University of Aarhus, Nordre Ringgade 1, DK-8000 Aarhus C, Denmark; 4 Department of Biochemistry and Molecular Biology, University of Southern Denmark, Campusvej 55, DK-5230, Odense M, Denmark; 5 Beijing Genomics Institute, Bei-shan Road, Shenzhen 518083, People's Republic of China; 6 Kunming Cell Bank, State Key Laboratory of Genetic Resources and Evolution, Kunming Institute of Zoology, Chinese Academy of Sciences, 32# Jiao-chang Road, Kunming, Yunnan 650223, People's Republic of China; 7 Wellcome Trust Sanger Institute, Wellcome Trust Genome Campus, Ickleton Road, Hinxton, Cambridge, CB10 1SA, UK

## Abstract

**Background:**

The regular mammalian X and Y chromosomes diverged from each other at least 166 to 148 million years ago, leaving few traces of their early evolution, including degeneration of the Y chromosome and evolution of dosage compensation.

**Results:**

We studied the intriguing case of black muntjac, in which a recent X-autosome fusion and a subsequent large autosomal inversion within just the past 0.5 million years have led to inheritance patterns identical to the traditional X-Y (neo-sex chromosomes). We compared patterns of genome evolution in 35-kilobase noncoding regions and 23 gene pairs on the homologous neo-sex chromosomes. We found that neo-Y alleles have accumulated more mutations, comprising a wide variety of mutation types, which indicates cessation of recombination and is consistent with an ongoing neo-Y degeneration process. Putative deleterious mutations were observed in coding regions of eight investigated genes as well as *cis*-regulatory regions of two housekeeping genes. *In vivo *assays characterized a neo-Y insertion in the promoter of the *CLTC *gene that causes a significant reduction in allelic expression. A neo-Y-linked deletion in the 3'-untranslated region of gene *SNX22 *abolished a microRNA target site. Finally, expression analyses revealed complex patterns of expression divergence between neo-Y and neo-X alleles.

**Conclusion:**

The nascent neo-sex chromosome system of black muntjacs is a valuable model in which to study the evolution of sex chromosomes in mammals. Our results illustrate the degeneration scenarios in various genomic regions. Of particular importance, we report - for the first time - that regulatory mutations were probably able to accelerate the degeneration process of Y and contribute to further evolution of dosage compensation.

## Abstract 2

The nascent neo-sex chromosomes of black muntjacs show that regulatory mutations could accelerate the degeneration of the Y chromosome and contribute to the further evolution of dosage compensation.

## Background

It is believed that in human and other eutherian mammals, the heteromorphic sex chromosomes evolved from a pair of ordinary autosomes between 166 and 148 million years ago [1-4]. After the birth of the sex-determining gene, extensive recombination suppression evolved between proto-X and proto-Y to prevent the sexual reversal along the entirety of the chromosome pair, with the exception of a short 'pseudoautosomal region' (PAR) [5]. Theoretical models predict that proto-Y chromosomes would further suffer a rapid accumulation of deleterious mutations and be subjected to drastic gene loss [6], consistent with only 45 genes surviving on the human Y chromosome as compared with 1,000 functional genes on the X [1,3,7]. Y degeneration is expected to be driven by multiple forces, including Muller's ratchet, background selection, the Hill-Robertson effect with weak selection, and the 'hitchhiking' of deleterious alleles by favorable mutations [5]. These factors can work on both ancient Y and heterosynaptic autosomes with suppressed recombination because of the diminished efficiency of natural selection along them. Newly evolved sex chromosome systems are required to test hypotheses about Y degeneration because ancient Y chromosomes bear few remaining traceable ancestral sequences. Such cases include the recently originated sex determination system, like that of *Silene *[8], or 'neo-sex chromosomes', formed through a recent fusion or translocation between an autosome and a sex chromosome followed by extensive recombination suppression, and thus showing inheritance patterns like that of the better known ancient sex chromosomes [9,10].

Our current knowledge of neo-sex chromosome evolution is primarily due to extensive work in *Drosophila *and plants [8,10-12]. Most of them focused on degeneration patterns in protein-coding regions of neo-Y alleles. However, given the elaborate regulation of gene expression [13,14], the scenarios of Y degeneration involving both gradual loss of functions of protein products as well as regulatory disorders remain to be elucidated. Furthermore, sex chromosome systems have evolved independently in different phyla many times, creating a need to examine their evolution in different taxa [15]. Sex chromosomes in mammals, including our own, have different autosomal origins from those of other organisms, involving entirely distinct gene sets in the process of sex determination and dosage compensation [15,16]. The inhibition of recombination between the mammalian proto-X and proto-Y was achieved through chromosomal inversions on the Y chromosome, whereas it was achieved through zero crossover in male germline cells in *Drosophila *[5,17]. Finally, different generation time and population size between mammals and other species would have great impact on the rate and patterns of mammalian Y-chromosome degeneration [18].

Thus, direct investigation of a neo-sex chromosome system in a mammal promises new insights into several fundamental issues, including both mode and tempo of mammalian Y degeneration, and how mammals cope with degenerated Y alleles before the creation of *Xist*-dependent dosage compensation [19]. In this study we sought to address these questions using the black muntjac (*Muntiacus crinifrons*), an Asian barking deer, as the model [20]. In this species, a male-specific extensive chromosome inversion on autosome 4 and fusion of its homolog to the ancient X [21,22] has led to the very recent creation (within the past approximately 0.5 million years) [23] of a neo-sex chromosome system, similar to the inferred creation of the ancient mammalian X-Y system. Such a rare nascent mammalian neo-sex chromosome system provides an unprecedented opportunity to study sex chromosome evolution in mammals.

## Results and discussion

### Neo-sex chromosomes in the black muntjac

**Figure 1:**
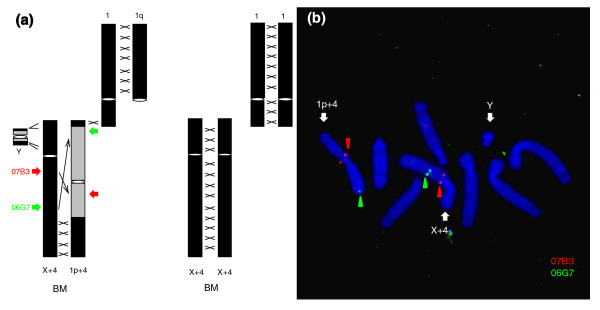
Neo-sex chromosomes of *Muntiacus crinifrons*. **(a) **Paring patterns in male and female black muntjacs during meiosis. The black areas on chromosomes represent homosynapsis regions, whereas gray areas represent heterosynapsis regions. Cross lines between chromosomes represent homologous recombination. A pentavalent involving five chromosomes will form during meiosis of male black muntjacs, whereas such structures are absent in female black muntjac. **(b) **Inversion on the neo-Y chromosome revealed by fluorescence *in situ *hybridization using two Indian muntjac bacterial artificial chromosome (BAC) clones, 06G7 (green) and 07B3 (red), as probes.

The black muntjac is endemic to a narrow region of southeastern China [20]. Habitat disruption in recent decades has rendered the species one of the most endangered mammals in the world. Cytogenetic analysis revealed a compact karyotype, with 2n = 8♀/9♂, and half of the genome forms a pentavalent (Figure 1a) during male meiosis [24]. The chromosome 4 pair has been shaped by several large chromosomal events. First, one copy experienced a centric fusion to the regular X chromosome, forming a new 'X+4' (see Additional data file 1). In addition, the short arm of chromosome 1 has undergone a male-specific translocation to the homologous chromosome 4, creating a primitive '1p+4' chromosome [21,22]. Surprisingly, subsequent inversions involving large part of the primitive 1p+4 were proposed to take place in male black muntjacs (regions from '22a' to '17a'; Additional data file 1) [21,22]. We further confirmed this chromosomal rearrangement using dual-color fluorescence *in situ *hybridization (FISH) with two bacterial artificial chromosome (BAC) clones of the Indian muntjac (*Muntiacus muntjac vaginalis*) as probes (Figure 1b). The green signal in the middle of X+4 has switched its location to the distal end of 1p+4 as a result of the inversion (Figure 1b). Because no inversion loop has been detected during meiosis of male black muntjacs [24], the whole 1p+4 should remain heterosynaptic except for the two distal ends, which are not involved in the inversion. These two ends are analogous to those of ancient human Y chromosome, which can still synapse with each other and can thus be regarded new 'PAR's. The X+4 chromosome can thus be regarded as a 'neo-X', and the 1p+4 as a 'neo-Y' chromosome, because of probable lack of recombination in the inverted region.

The black muntjac X+4:1p+4 neo-sex chromosome pair comprises approximately one-fifth of the entire genome and thus bears thousands of neo-sex gene pairs [21,24]. Although X-autosome fusions were also detected in other muntjac species, such 1p+4 inversion is found exclusively in male black muntjacs [25]. The absence of such a system within *Muntiacus *genus, from which black muntjac is estimated to have diverged within the past 0.5 million years [23], indicates a very recent origin, and makes this system the youngest known mammalian neo-sex systems.

### Variation pattern in neo-Y noncoding regions

To seek molecular evidence for inhibition of recombination and to compare the evolutionary patterns of neo-X and neo-Y chromosomes, we sequenced the intergenic and intronic fragments from the inverted region composing a total of 35.1 kilobases (kb), as well as 12.6 kb from the new 'PAR's and autosomal regions for two male and one female black muntjacs, as well as the orthologous segments from one male Indian muntjac, which is taken as the outgroup.

**Figure 2:**
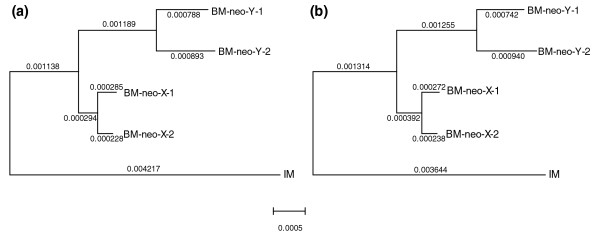
Dendrogram constructed using noncoding sequences of neo-Y and neo-X fragments. A total of 35.1-kilobase noncoding sequences of neo-Y and neo-X alleles in the black muntjac and the orthologous sequences in the Indian muntjac as the outgroup were used to construct the dentrogram. 'BM' stands for 'male black muntjac'; 'IM' stands for 'Indian muntjac'; '1'and '2' represent the two male black muntjac individuals separately. **(a) **Tree constructed by neighbor-joining method. Branch lengths calculated using Kimura's two-parameter method are shown above the corresponding branches. **(b) **Tree constructed by maximum likelihood method. Branch lengths calculated by baseml in PAML package using 'HKY85' method were shown above the corresponding branches.

Under neutrality, intraspecific genetic diversity (*θ*) is expected to be proportional to the effective population size (*N*_e_) times the mutation rate (*μ*; specifically, *θ *= 4*N*_e_*μ*) [26]. Therefore, if recombination has authentically ceased between neo-Y and neo-X, then we would expect a reduced DNA polymorphism caused by reduced *N*_e _for the neo-Y regions [6,27]. Otherwise, the polymorphism level of neo-Y should be similar to that of 'PAR' regions or autosomes. Consistent with the former expectation, we found a significantly lower number of segregating sites (χ^2 ^test, *P *< 0.01; see Materials and methods [below]) and a lower polymorphism level of neo-Y regions (0.00168 ± 0.00120 versus 0.00190 ± 0.00029) compared with regions that undergo homologous meiotic pairing (Table 1), indicating suppression of recombination between neo-Y and neo-X. Of course, other factors including the Hill-Robertson effect, background selection, hitchhiking effect, or Muller's ratchet could have all reduced the polymorphism level within the investigated noncoding regions [6]. In contrast, the 'male-driven evolution' effect, which proposes that the neo-Y would undergo more rounds of cell division per generation in male than in female germlines, would increase the mutation rate and polymorphism level of neo-Y alleles compared with those of neo-X and autosomes [28]. Our phylogenetic analysis of neo-X and neo-Y sequences in the male black muntjac confirms that the neo-Y alleles have accumulated far more mutations than neo-X (Figure 2; statistically significant, by Tajima's relative rate tests, *P *< 0.001).

**Table 1:** Segregating sites and polymorphism in noncoding sequences of different genomic regions in two male black muntjacs

	neo-Y regions	PAR/autosomal regions
Sequenced length (bp)	35,156	12,653
Segregating sites^a^	59	44
Segregating sites/kb	1.68	3.48
Polymorphism (*θ*_w_)	0.00168 ± 0.00120	0.00190 ± 0.00029

^a^Segregating sites means total sites with substitutions in the investigated alleles. Standard deviations calculated based on variance (see Materials and methods) were shown for polymorphism data. bp, base pairs; kb, kilobases; PAR, pseudoautosomal region.

A direct estimate of the degree of male-driven evolution effect (*α*) on neo-Y can be derived from the comparison of branch lengths of homologous neo-Y and neo-X. We calculated the ratios of neo-Y to neo-X mutations separately from internal, external, and summed branch lengths of neo-Y and neo-X, and we further estimated the *α *values based on the method proposed by Miyata and coworkers [29] (Table 2; see Materials and methods [below]). Because all of the ratios are higher than 3, the *α *values estimated from different branches all approach to infinity [29]. This indicates a strong male-driven effect on the neo-Y chromosome in the male black muntjac, and a small effect of ancestral polymorphism on the estimate [29-31]. It has been reported that great variation in mutation rates in different genomic regions greatly affect the estimation of *α *value [32]. However, such effects may be limited in our case because the investigated regions are randomly sampled along the neo-Y chromosome and then concatenated together to represent a chromosome-wide estimate (see Materials and methods [below]). Male-specific methylation effects in the germline may also make little contribution to such differences in mutation rate between neo-X and neo-Y alleles. It predicts a greater GC content on neo-X alleles and an elevated rate of C → T transitions on neo-Y alleles [33]. However, we detected a similar GC content and an almost identical ratio of GC → AT versus AT → GC substitutions between the neo-Y and neo-X alleles (Additional data file 2).

**Table 2:** Estimation of *α *(male:female ratio of mutation rate) from different branch lengths in the neo-sex system of black muntjac

	neo-Y/neo-X	*α*	95% CI of *α*
Internal branches	4.0442 ± 1.2633	∞	25.3848 to ∞
External branches	3.2768 ± 1.2356	∞	4.2578 to ∞
Summed branches	3.6857 ± 0.8867	∞	27.8507 to ∞

Note that we calculated neo-Y:neo-X ratios of mutation rate from branch lengths designated in Figure 2a. We further calculated their confidence intervals (CIs) and estimated *α *values following [30,31].

The net effect of all of these factors explains why the polymorphism of neo-Y region (0.00168) is one-quarter higher than that of recombining regions (0.00190/4; Table 1). Finally, other factors, including sexual selection and population subdivisions, could have effects on the polymorphism pattern [34]. These findings indicate that it is highly likely that recombination has ceased between the inverted neo-Y region and its homolog.

### Degeneration in coding regions of neo-Y-linked genes

Because of the limited sample size of neo-Y alleles, the polymorphism data exhibit a high degree of variance, and differences between recombining and nonrecombining noncoding regions may only be indicative rather than statistically significant (Table 1). That stated, we continued to seek for other typical evidence of recombination inhibition (accumulation of deleterious mutations due to reduced efficiency of natural selection) [6]. To evaluate the degeneration effects of such mutations on neo-Y-linked genes, we characterized 23 gene pairs that are located in the inverted region on the neo-Y chromosome (Additional data file 1), one pair in the neo-Y 'PAR' region, and six pairs on autosomes. These genes were selected based on the annotations of BAC sequences of the Indian muntjac and cow genomic information in order to represent a diversity of regions across the neo-sex chromosomes [35]. In total, we obtained about 25 kb cDNA sequences in the inverted region and 6.3 kb in noninverted regions from two male black muntjacs, one female black muntjac, and one male Indian muntjac.

Among the 23 gene pairs located in the inverted region, we observed 14 mutations in eight genes shared by the two males in the protein-coding or untranslated regions (UTRs) of the neo-Y alleles (Table 3). In contrast, we detected no variation in genes located in the neo-Y 'PAR' region. For the autosomal region, only one out of six genes is heterozygous at one site in both the two male black muntjacs. Such an elevated rate of gene evolution exhibited by neo-Y alleles relative to their neo-X homologs and other autosomal genes is consistent with reduced efficiency of natural selection against deleterious mutations in the neo-Y. Combined with the variation patterns in intronic and intergenic regions (see the section above), these findings confirmed the recombination inhibition on the neo-Y chromosome.

**Table 3:** Summary of neo-Y specific mutations occurred in the cDNA of investigated genes

Gene symbol	Positions on neo-Y^a^	Shared mutation	Polymorphic mutation
*MYO1D*	17a		One synonymous substitution
*RPL19*	17a	One synonymous substitution	One synonymous substitution
*CACNB1*	17a		**A to S**^b^
*CLTC*	17a	Two synonymous substitutions	
*ZNF24*	17a	One synonymous substitution	
*AKAP7*	3b	Three synonymous substitutions; V to M	
*SYNE1*	3b	One synonymous substitution; I to V; **V to E**^b^	Two synonymous substitutions
*SNX22*	8	46 bp deletion in 3'-UTR	
*RIC8B*	1a	**S to P**^b^	
*SCN1A*	3a		**D to G**^b^
*MPPE1*	4a	One synonymous substitution	

^a^Positions correspond to chromosomal regions depicted in Figure 1a. ^b^Missense mutations with drastic change of biochemical property of amino acid (polarity or charge), and those in bold represent sites that are highly conserved in other eutherian mammals with published sequences. We defined 'shared mutations' as those mutations detected in neo-Y alleles in both male black muntjacs, whereas 'polymorphic mutation' are those detected in only one male black muntjac.

Specifically, four nonsynonymous substitutions shared by the two male black muntjacs and two polymorphic nonsynonymous substitutions in one of the males were observed (Table 3). Of these six changes, four are highly conserved in amino acids across six other eutherian mammals (human, chimpanzee, dog, mouse, rat, and cow; Table 3). These four mutations change the polarity or charge of the amino acid, which might severely affect the protein function of the neo-Y copies. We also detected a neo-Y-linked deletion in 3'-UTR of the gene *SNX22 *in both males investigated. This deletion abolishes a microRNA target site (see below) and thus may affect stability of the neo-Y allele's mRNA [36,37]. By taking into account the above-described four possible deleterious nonsynonymous mutations and the deletion in the 3'-UTR of *SNX22*, we conservatively estimate the rate of accumulation of deleterious mutations in exon regions of neo-Y genes to be approximately 0.4 mutations/kb per million years (5 mutations/25 kb per 0.5 million years). We also estimated that only about 1.3 nonsense mutations would be expected within 25 kb of investigated cDNA sequences, assuming equal chance of mutation at each site (see Materials and methods [below]). Such a low probability of nonsense mutation plus the effect of possible purifying selection give credence to our observation that there were no coding frame-disrupting mutations among the investigated muntjac neo-Y alleles.

This finding differs from that of a recent investigation conducted in the neo-sex system of *Drosophila miranda *[38], which found that the neo-Y alleles of 24 out of 64 genes contain premature stop codons and/or frameshift mutations, suggesting an average loss rate of 20 genes per million years in the proto-Y of *Drosophila*. Such a drastic difference in the degeneration rate between neo-Y chromosomes of mammals and *Drosophila *could be attributable to the great differences in their mutation rates (2.2 × 10^-9 ^versus 3.1 × 10^-7 ^per base per year for mammal and *Drosophila*, respectively) [39,40], generation time, DNA repair efficiencies [41], and effective population size [18]. It also suggests that, during the nonlinear process of mammalian Y degeneration [3], the rate of gene loss might be very slow in the early stage of mammalian Y chromosome evolution.

Multiple factors can fix the above neo-Y specific mutations in populations in both coding and noncoding regions, such as Muller's ratchet, background selection, hitchhiking effects on linked deleterious mutations, and faster mutation rate in males [6,28,42,43]. Muller's ratchet must have had strong effect on accumulation of these mutations, given that the number of mutant-free chromosomes in a population is positively correlated with the effective population size (*N*_e_) [6,18]. Because of the usual small *N*_e _values of mammals, mutant-free neo-Y alleles should be vulnerable to an irreversible loss by random drift by the 'ratchet' process, which further leads to accumulation of deleterious mutations. It is less likely that the observed neo-Y variations were from either ancestral polymorphism or gene conversion between neo-X and neo-Y, because we used both female black muntjac and Indian muntjac genes as the references to define neo-Y mutations [42]. It is also less likely that there were recent selective sweeps on the neo-Y of the black muntjac, based on the DNA polymorphism data. Recent strong positive selection would homogenize the neo-Y chromosomes among individuals [6], but we observed many polymorphic sites existing in both noncoding and coding regions of the muntjac neo-Y chromosome (Tables 1 and 3). Regarding other processes, because of the difficulty associated with collecting population data for such a rare species, we cannot currently test the existence of either a slower rate of adaptive evolution [44] or background selection [6], as previously proposed for *D. miranda *[10].

### Degeneration in *cis*-regulatory regions of neo-Y-linked genes

Recent studies have revealed that *cis*-regulatory regions more often underlie the expression divergence between species and evolution of morphologic diversity [14,45,46]. Apart from the direct evidence of degeneration in coding regions presented above, we sought to survey degeneration in regulatory regions on the neo-Y chromosome. We analyzed putative promoters expanding 1.5 kb around transcriptional start sites (TSSs) of eight neo-Y genes, which have mutations shared by the two male black muntjacs in their protein-coding/UTR regions (Table 3).

**Figure 3:**
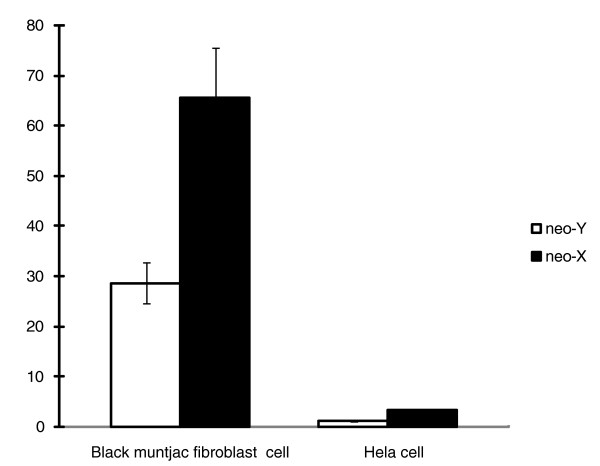
Mutation in the promoter of *CLTC *gene severely causes downregulation of the neo-Y copy. Dual-reporter assay of promoter activities of *CLTC*. Standard error among triplicates was shown on the bar.

We detected neo-Y specific mutations shared by both males present in the investigated regions in three out of eight genes (see Additional Data File 3). In order to assess the effect of these mutations on gene expression, allelic promoters were subsequently cloned into pGL3-Basic plasmid in front of a firefly luciferase reporter gene. Each plasmid was mixed with a plasmid (pRLs-TK) containing a constitutive promoter driving sea-pansy luciferase reporter gene and co-transfected into Hela cells and male black muntjac fibroblast cells. The ratios of firefly luciferase to sea-pansy luciferase, representing normalized promoter activity, were compared between neo-Y-linked promoters and neo-X-linked promoters. We found a significant decrease in neo-Y promoter activity in both *Hela *and black muntjac cell lines for the *CLTC *gene (Figure 3). We did not detect promoter activities for the cloned fragments of other two genes; specifically, there is no detectable expression of the reporter genes using the cloned fragments as the promoters. The only detected neo-Y specific mutation is a 1 base pair (bp) insertion 55 base pairs upstream of the putative TSS of the *CLTC *gene shared by both males (Additional data file 3). It is probably the mutation responsible for reducing the transcription level of neo-Y, given that this region was proposed to contribute positively to the core promoter activity by a recent comprehensive analysis of 387 promoter structures in humans [47].

In addition to mutations in promoter regions, we also detected a neo-Y specific deletion shared by both male black muntjacs in the 3'-UTR of the gene *SNX22 *as mentioned above (Table 3). The abundance of microRNA targeted sequences in this region drove us to investigate whether this deletion would affect such motifs [36,37]. Using human microRNA database as a reference, we found that the deleted region on the neo-Y is in fact predicted to be a binding motif targeted by the microRNA hsa-mir-210 [48], which also has orthologs in cow and mouse with validated expression. Intriguingly, we found evidence that this same microRNA targeted motif is also deleted because of male-specific RNA editing in intact neo-X alleles (see Additional data file 4); 23 bp overlapping the target sequence are absent from transcripts in males (the edited form is much less frequent in females; see Additional data file 5). The diminutive size of the involved sequence and lack of splicing boundaries suggest that it is a true RNA editing event rather than a splicing event. It could be a result of selection against allelic expression imbalance between neo-X and neo-Y or a sign of degeneration of *trans *factors on neo-Y in males, which control the RNA-editing degree in the 3'-UTR of *SNX22*.

One of the fundamental issues in the process of Y-chromosome degeneration is whether it is mainly driven by direct degradation of the protein products (for example, in *D. miranda*) or reducing gene expression levels on an evolving Y [6]. Our results suggest that these two processes may work in concert after the immediate recombination suppression on the neo-Y chromosome. It is proposed that deleterious mutations with mild fitness effects have a higher rate of fixation on a degenerating Y [6], as illustrated in Table 3. After accumulation of such mutations in the protein coding regions, a subsequent reduced expression of Y would be favored by natural selection to prevent the production of defective Y-linked products [6,18]. Alternatively, reduced expression of Y may take place before the accumulation of deleterious mutations in protein products. In the case of *CLTC *and *SNX22*, both are broadly expressed genes in most of the human tissues, with orthologs present in all vertebrates whose genomes have been sequenced. *CLTC *plays an critical role through coating membrane vesicles during endocytosis [49] and *SNX22 *is a member of the protein family responsible for protein trafficking [50]. The protein coding regions of these housekeeping genes must be under strong selective constraints, in which no amino acid replacement substitution was detected (Table 3). On the other hand, recent studies showed that *cis*-regulatory regions - especially microRNA and transcription factor binding sites - usually have lower interspecies sequence conservation compared with protein coding regions, suggesting that regulatory mutations can be more easily fixed [46,51].

This suggests a scenario involving two independent steps of degeneration. First, mutations in regulatory regions (for example, the promoter of *CLTC*) would decrease or even turn off the gene expression of the Y alleles. Second, decreased expression could reduce selective constraints acting on the protein products of proto/neo-Y-linked genes [52]. We note that both these processes could in fact be favored to maintain the optimal gene dose between males and females after the establishment of dosage compensation in X [53]. Also, both processes would further accelerate the degeneration process in the Y-linked coding regions, which is supported by the recent finding that lower expressed neo-Y genes appear to have a faster accumulation rate of deleterious mutations in *D. miranda *[54].

### Transcription divergence between neo-Y and neo-X alleles

**Figure 4:**
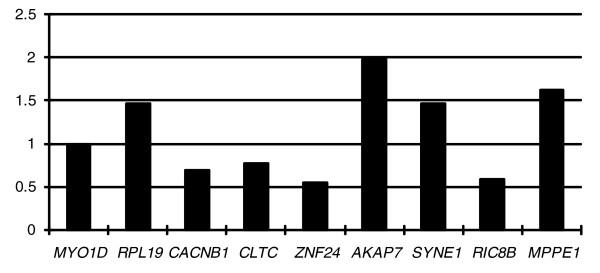
Expression divergences between nine neo-Y and neo-X gene pairs. All expression assays were done in duplicate and double checked in both male individuals. Mean expression ratios of neo-Y to neo-X are shown. The genes are arranged following the order from the centromere to the distal region of the 1p+4 chromosome.

We semi-quantified and compared the mRNA abundance of neo-Y and neo-X alleles in 11 genes with mutations listed in Table 3. Consistent with our promoter assay, *CLTC *exhibits a lower expression level on neo-Y. We found significant expression difference between neo-Y and neo-X allele only in the gene *SNX22 *(Fisher's exact test, *P *< 0.01). However, this gene was further excluded from the result, together with *SCN1A*, because of their effects of allelic-biased amplification (see Materials and methods [below]). Eight of the remaining nine genes exhibited differential expression; interestingly, as many showed higher expression on neo-Y as on neo-X (4 versus 4; Figure 4). This finding suggests that neo-Y alleles can be distorted from normal expression level, either downregulated or upregulated, as a result of degenerating control of gene expression. Together with the mutation analysis results presented above, the expression patterns of the nine investigated genes exhibit a similar random inactivation mode of gene evolution on a degenerating Y chromosome proposed for *D. miranda *[12]. This model predicts that neo-Y genes are randomly inactivated, regardless of their level of adaptation. As shown in Figure 4, the regulation direction of neo-Y genes fluctuates along the neo-Y chromosome, and between some adjacent genes it is even opposite, suggesting that there is no large segment inactivation in the black muntjac's neo-Y chromosome. Two genes (*CLTC *and *ZNF24*) with only synonymous neo-Y mutations exhibited lower expression level, whereas two genes (*SYNE1 *and *AKAP7*) with nonsynonymous neo-Y mutations exhibited higher expression level. In addition, the human orthologs of all nine investigated genes are widely expressed housekeeping genes. Therefore, whether a neo-Y allele is subjected to expression alteration or the direction of alteration may be not strongly correlated with the characteristics of the gene.

This random inactivation pattern indicates that the *Xist*-dependent dosage compensation[19] has not spread across the whole neo-X chromosome in females within the past 0.5 million years. This could be attributed to a lack of sufficient time for the establishment of action of *Xist *on neo-X regions. Concordantly, there is also clear molecular evidence that neo-X chromosome in male *D. miranda *is only partially dosage compensated [55]. A recent study [56] showed that even in the 'ancient' sex determination system, such as that of human, 15% of X-linked genes would escape the X inactivation, suggesting that the mammalian dosage compensation mechanism is leaky and its evolution process might be slow. As suggested by our results in male black muntjacs described above, not all the neo-Y alleles have been subjected to the reduction of gene expression. A nonspecific whole-set dosage compensation mechanism is likely to be seriously deleterious for genes that are transcriptionally active or are undergoing degeneration. It is also possible that a major regulator of dosage compensation, such as *Tsix *gene, counteracted *Xist *RNA on neo-X regions to prevent such deleterious effects [57,58]. However, such delicate regulation is less likely to have been established within such a short evolutionary time. Overall, we propose here that in the early stages, dosage compensation in males might have evolved gradually in a gene-by-gene fashion. This model is also consistent with the conjecture that evolution is accomplished more through small steps than large changes [59].

Charlesworth [18] has proposed two models to describe evolution of dosage compensation in manners similar to the two paths of Y-chromosome degeneration mentioned above. The common features of both models involve the upregulation of gene expression from the proto-X chromosome at the initial stage of Y degeneration [18], which has been suggested by recent global expression analyses conducted in *Drosophila*, worm, and mammals [60,61]. One model proposed by Charlesworth suggests that there would be selection promoting upregulation of the transcription of X alleles to compensate for defective products, with mutations accumulated in the coding regions of Y alleles. Alternatively, mutations could arise to make the Y alleles less responsive to the regulatory molecules, as exemplified by the case of *CLTC *in this study. It would consequently be a passive process, upregulating the X alleles' expression as a result of the excess of regulatory molecules from the Y alleles [18]. Further study into the paths of Y chromosome degeneration and comparison of neo-X expression between males and females using more gene pairs would be able to uncover which model mainly contributes to the Y degeneration and evolution of dosage compensation.

## Conclusion

Here we characterize the recently formed neo-sex system of the black muntjac. This unprecedented system is valuable for studying mammalian Y-chromosome degeneration and evolution of dosage compensation. Our results provided molecular evidence for recombination suppression in the neo-Y chromosome. As a result, excess of putatively deleterious mutations were observed in the coding regions of the investigated genes, probably because of the Muller's ratchet effect or background selection. Most importantly, we report here the first study of the role of regulatory mutations during the degeneration process of mammalian Y, and we provide empirical data showing their degenerative effect on gene expression. Such mutations might further accelerate the degeneration and give rise to the evolution of dosage compensation in a gene-by-gene manner. These results demonstrate that mammalian Y degeneration is a complex gradual process spanning diverse genomic structures.

## Materials and methods

### FISH, separation of 1p+4 by cell Sorter, PCR, and sequence analysis

Frozen kidney from a male black muntjac, and fibroblast cell lines of another male (KCB82001) and a female black muntjac (KCB81002E) were provided by Kunming Cell Bank of the Chinese Academy of Sciences. Total genomic DNA was extracted using the PURRGENE^® ^DNA Isolation Kit (Gentra Systems Inc., Minneapolis, MN USA), and total RNA was extracted using RNeasy^® ^Mini Kit (Qiagen, Valencia, CA USA). Based on the syntenic relationship among black muntjac, Indian muntjac, and cow and annotation results of Indian muntjac [35], we selected genes randomly distributed along the neo-Y chromosome, PAR region, and autosomal regions for primer designs (Table 3). The investigated noncoding regions are introns or flanking intergenic regions of these selected genes. We prepared cell suspensions of the male black muntjac for further FISH and flow sorting of 1p+4 chromosomes. The sorting procedure using a FACStar Plus flow sorter (Becton Dickinson, Franklin Lakes, NJ USA) and FISH analysis using Indian muntjac BAC clones (06G7 and 07B3) as probes were performed as previously described [21].

We used two strategies to discriminate neo-X and neo-Y alleles. First, we compared genomic DNA PCR products of Indian muntjac, and female and male black muntjacs, and we inferred neo-Y-specific alleles. We also used flow-sorted 1p+4 chromosomes as PCR templates to confirm the neo-Y-specific mutations. The products were subject to sequencing with BigDye Terminators v3.0 (Applied Biosystems) after purification (QIAquick^® ^PCR Purification Kit; Qiagen, Valencia, CA USA). Trace data were manually trimmed and aligned from both directions using Lasergene suite (DNASTAR Inc., Madison, WI USA). We concatenated sequences of noncoding regions together for further analysis. Segregating sites in these regions were counted and polymorphism data were analyzed using DnaSP 4.0 [62]. Confidence intervals for polymorphisms were calculated by variance [63].

If we assume the 1p+4 chromosome can recombine with its homologous chromosome, then its polymorphism level is expected to be similar to that of other autosomes or 'PAR' regions. Under such a null hypothesis, we test the significance of difference for polymorphism between 1p+4 and other chromosome regions with χ^2 ^test. Sequences of neo-Y and neo-X alleles were subjected to construction of phylogenetic trees and distance calculation with MEGA 3.1 [64]. After removing regions with indels, gene tree was constructed using the neighbour joining and maximum likelihood method, and distances were calculated with Kimura's two-parameter and HKY85 model, respectively [65].

We used internal, external, and summed branch lengths in Figure 2a to estimate *α*. Comparison of *α *estimates from different branch lengths can be used to test whether the ancestral polymorphism have affected the calculation [31]. According to the method proposed by Miyata and coworkers [29], Y/X = 3*α*/(2 +*α*), where Y and X stand for mutation rate for Y-linked and X-linked sequences, respectively. The variance of Y is V(Y) = Y(1 - Y)/(L [1-4Y/3]^2^) and the variance of X is V(X) = Y(1 - X)/(L [1-4X/3]^2^), where L is the length of the sequence [31]. The variance of Y/X is V(Y/X) = V(Y)/E(X)^2 ^+ E(Y)^2^V(X)/E(X)^4^. The 95% confidence interval of *α *was estimated, following the method proposed by Huang and coworkers [30]. Because Y/X(0.001189/0.000294, internal branches) is greater than 3 based on branch lengths of neo-Y and neo-X in this study, the *α *value approaches infinity. To estimate expected stop condon number *E*(*S*) in the investigated coding regions, we assume that only one substitution would arise per codon, given the extremely young age of this neo-sex system. Assuming the chances of mutation are equal across the sequences, *E*(*S*) would be given by the following: $E(S)=3\mu t\sum_{i=1}^{n}p_{i}$, where *μ *is the estimated mammalian mutation rate measured per base [39], *t *is the age of the neo-sex system, *n *is the number of the codon in the investigated region and *p*_i _is the probability of a certain codon becoming a stop codon.

### Promoter assay

We predicted the TSS information for black muntjac genes with ClustalW [66], using orthologous human, mouse, and cow sequence as reference. Regions expanding 1 kb upstream and 500 bp downstream of the predicted TSS were amplified as putative promoter regions in black muntjacs. The PCR products were subject to TA cloning using pMD19-T vector (TaKaRa, Dalian, Shangdong China) for screening products without PCR artifacts and further enzyme digestion. The digested PCR product was cloned into pGL3-Basic firefly luciferase vector (Promega, Madison, WI USA) using T4 ligase (Invitrogen, Carlsbad, CA USA). Then the vector was transformed into competent cell (TOP10; Invitrogen, Carlsbad, CA USA) and purified by QIAprep^® ^Spin Miniprep Kit (Qiagen, Valencia, CA USA).

Hela and male black muntjac fibroblast cell lines were respectively seeded into 24-well plates to reach densities of 10^4 ^to 10^5 ^cells per well 24 hours before transfection. Generally, 0.2 μg pGL3-Basic vector with cloned promoter and 0.04 μg *Renilla *(sea-pansy) luciferase vector (pRL-TK; Promega, Madison, WI USA) were mixed in Opti-MEM^® ^I Reduced Serum Medium (Invitrogen, Carlsbad, CA USA) and co-transfected into cells with Lipofectamine™ 2000 (Invitrogen, Carlsbad, CA USA). We used pGL-Basic vector as negative control and assayed the luciferase signal with the Dual-Luciferase^® ^Reporter 1000 Assay System (Promega, Madison, WI USA). The ratios of firefly luciferase to sea pansy luciferase were compared between neo-Y-linked promoters and neo-X-linked promoters. All of the assays for each promoter were done in triplicate.

### Expression abundance analysis

We amplified coding regions containing neo-Y-linked mutations from genomic DNA and total RNA (One Step RNA PCR Kit, TaKaRa, Dalian, Shangdong China), respectively. The genomic PCR was used as a control to exclude PCR biases caused by allelic preferential amplification. Out of 11 genes, we observed biased amplification for only two genes, namely *SCN1A *and *SNX22*; the extent of the amplification bias is similar to the copy abundance difference observed in the mRNA assay. The reaction was done for 25 to 30 cycles, and the PCR products were cloned into pMD19-T vector (TaKaRa, Dalian, Shangdong China). Twenty to 30 white clones were randomly picked and were subject to further sequencing. The expression ratio of neo-Y and neo-X alleles was determined by counting the number of separate alleles. All assays were conducted in duplicate and double checked in both male individuals. We further compared the mean expression ratio of duplicates for different genes.

## Abbreviations

BAC, bacterial artificial chromosome; bp, base pair; FISH, fluorescence *in situ *hybridization; kb, kilobases; *N*_e_, effective population size; PAR, pseudoautosomal region; PCR, polymerase chain reaction; TSS, transcriptional start site; UTR, untranslated region.

## Competing interests

The authors declare that they have no competing interests.

## Authors' contributions

WW and FY designed the project, analyzed the data, and wrote the paper. QZ, JW, and LH performed the PCR experiments, and expression and promoter assays. WN, JW, YL, and XZ cultivated the fibroblast cells and performed the FISH experiments. QZ and JW analyzed the data and wrote the paper. All authors read and approved the final manuscript.

## Additional data files

The following additional data are available with this paper. Additional data file 1 is a schematic ideogram showing the neo-sex system of *Muntiacus crinifrons*. Additional data file 2 is a description with a table showing substitution patterns on noncoding sequences of neo-Y and neo-X. Additional data file 3 is a table showing neo-Y linked mutations in promoter regions and their effects on allelic expression. Additional data file 4 is a figure showing the RNA editing process of neo-X allele in 3'-UTR of *SNX22*. Additional data file 5 is a table showing **t**he RNA editing degree of *SNX22 *neo-X allele in male black muntjacs is higher than that in female.

## Supplementary Material

Additional data file 1 Numbers beside the chromosomes represent homologous chromosomes of *Muntiacus reevesi *defined by comparative chromosomal painting [21]. Only the proximal region 17b and the distal region below 17a can form homologous pairing and thus recombine with their homologs. Regions spanning from '22a' to '17a' cannot recombine because of a large intrachromosomal inversion. Regions '8', '17a', and '17b' were derived from short arm of autosome 1 (1p) and further experienced rearrangements, forming the current order along 1p+4. We also showed location information for the investigated 23 genes [22,35]. Click here for file

Additional data file 2 Presented is a description with a table showing substitution patterns on noncoding sequences of neo-Y and neo-X. Click here for file

Additional data file 3 Presented is a table showing neo-Y linked mutations in promoter regions and their effects on allelic expression. Click here for file

Additional data file 4 The UTR region is designated in lower case, whereas the protein-coding region is presented in uppercase. Splicing is unlikely because there is no splicing signal at the boundaries of deleted region and an intron shorter than 30 bp is scarce in mammals. Click here for file

Additional data file 5 Presented is a table showing **t**he RNA editing degree of *SNX22 *neo-X allele in male black muntjacs is higher than that in female. Click here for file
