# Supplementary figures and images for: Neo-sex chromosomes in the black muntjac recapitulate incipient evolution of mammalian sex chromosomes
Source: Genome Biol. 2008 Jun 14;9(6):R98. doi: 10.1186/gb-2008-9-6-r98 (PMC2481430; doi:10.1186/gb-2008-9-6-r98)

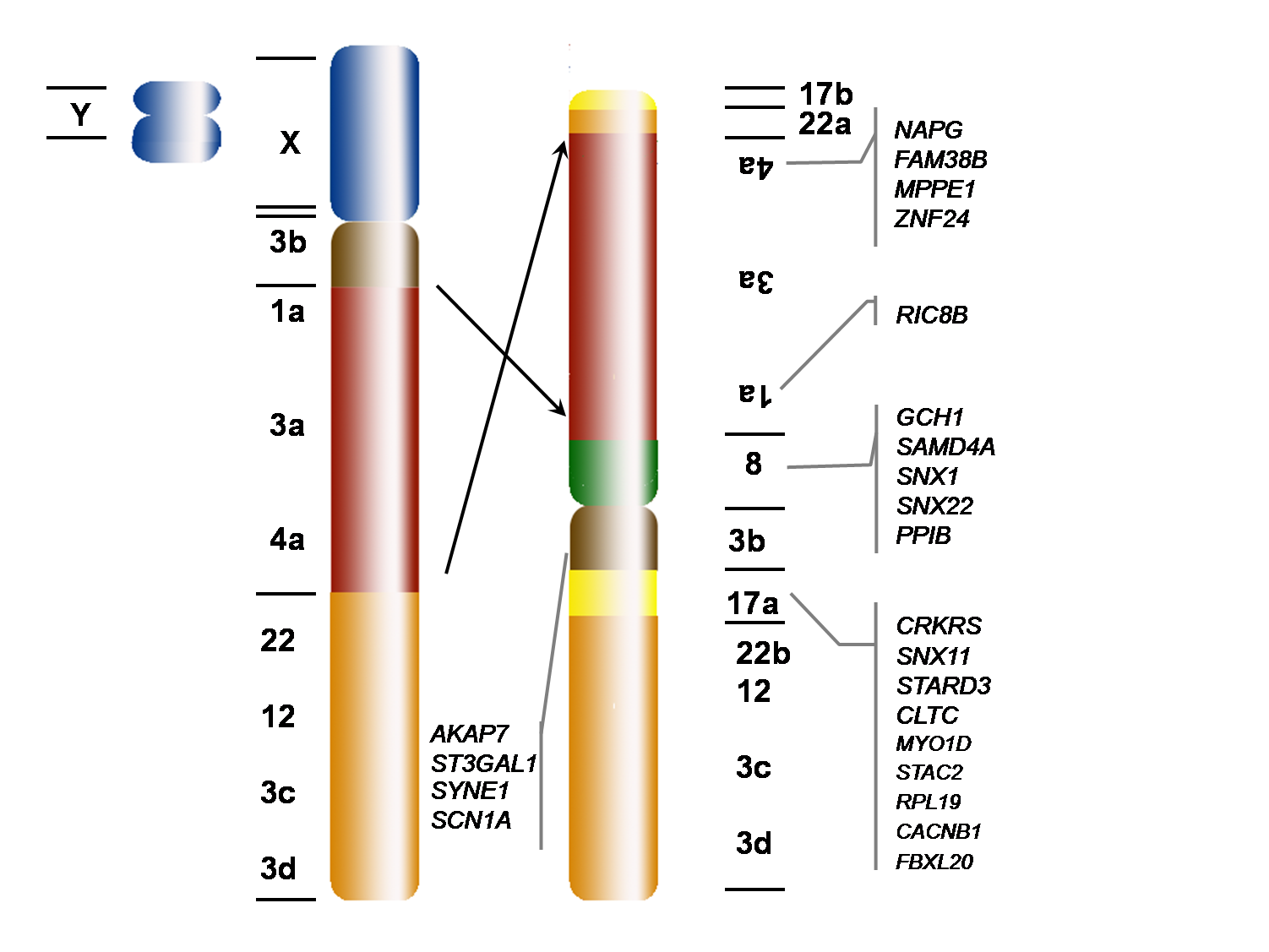

Supplement: Additional data file 1 — Numbers beside the chromosomes represent homologous chromosomes of Muntiacus reevesi defined by comparative chromosomal painting [21]. Only the proximal region 17b and the distal region below 17a can form homologous pairing and thus recombine with their homologs. Regions spanning from '22a' to '17a' cannot recombine because of a large intrachromosomal inversion. Regions '8', '17a', and '17b' were derived from short arm of autosome 1 (1p) and further experienced rearrangements, forming the current order along 1p+4. We also showed location information for the investigated 23 genes [22,35]. [file gb-2008-9-6-r98-S1.tiff]

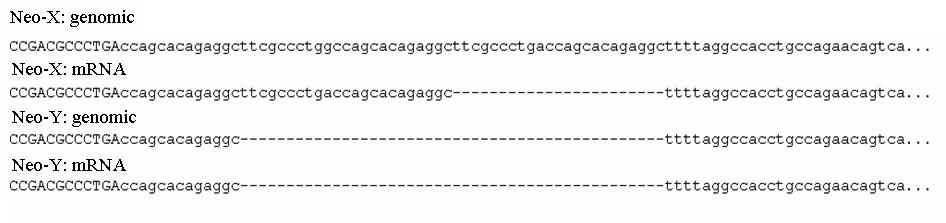

Supplement: Additional data file 4 — The UTR region is designated in lower case, whereas the protein-coding region is presented in uppercase. Splicing is unlikely because there is no splicing signal at the boundaries of deleted region and an intron shorter than 30 bp is scarce in mammals. [file gb-2008-9-6-r98-S4.tiff]
