# Supplementary material for: Neo-sex chromosomes in the black muntjac recapitulate incipient evolution of mammalian sex chromosomes
Source: Genome Biol. 2008 Jun 14;9(6):R98. doi: 10.1186/gb-2008-9-6-r98 (PMC2481430; doi:10.1186/gb-2008-9-6-r98)
Supplement: Additional data file 2 — Presented is a description with a table showing substitution patterns on noncoding sequences of neo-Y and neo-X. [file gb-2008-9-6-r98-S2.doc]

We detected average GC content of neo-Y alleles (41.895%) is only slightly lower than their neo-X counterparts (41.905%), suggesting male-specific methylation have limited/no effects on the mutational bias [33]. To be specific, the proportion of GC→AT vs. AT→GC ratio is almost identical on neo-Y and neo-X alleles (1.03 vs. 0.95, Table 1). No significant heterogeneity between these two classes of substitutions were observed (Fisher’s exact test, *P*=0.25). We also estimated kappa (transition/transversion ratio) value for neo-Y and neo-X separately compared with outgroup using PAML[63]. The average kappa for neo-Y is 3.82165, while neo-X is 3.8413. All these data suggest the effect of mutational bias caused by sex difference is limited in such young neo-sex system.

Table 1. Numbers of substitutions from neo-Y and neo-X linked non-coding sequences.

|  | GC→AT | AT→GC | Others |
| --- | --- | --- | --- |
| Neo-Y | 111 | 108 | 54 |
| Neo-X | 77 | 81 | 32 |

Substitutions were counted compared to homologous sequences of Indian muntjac. ‘Others’ represent changes between ‘G↔C’ or ‘A↔T’.
