# Supplementary material for: Neo-sex chromosomes in the black muntjac recapitulate incipient evolution of mammalian sex chromosomes
Source: Genome Biol. 2008 Jun 14;9(6):R98. doi: 10.1186/gb-2008-9-6-r98 (PMC2481430; doi:10.1186/gb-2008-9-6-r98)
Supplement: Additional data file 3 — Presented is a table showing neo-Y linked mutations in promoter regions and their effects on allelic expression. [file gb-2008-9-6-r98-S3.doc]

Summary of neo-Y linked mutations in promoter regions and their effects on allelic expression

| Gene Symbol | Position | Mutation | Fluorescence value (Neo-Y/Neo-X) | |
| --- | --- | --- | --- | --- |
|  |  |  | Hela | Muntjac Fibroblast |
| *CLTC* | -55bp | 1bp insertion | 28.70/65.58 | 1.136/3.293 |
| *SNX22* | -637bp | 5bp insertion | - | - |
| *SYNE1* | -248bp | A to G | - | - |

The positions of promoter mutations on neo-Y chromosome were defined as the distance to the TSS site corresponding to that of cow. And the normalized fluorescence value of neo-Y and neo-X in investigated cell lines were shown. The tested putative promoters for *SNX22* and *SYNE1* showed no promoter activity.
