# Supplementary material for: Neo-sex chromosomes in the black muntjac recapitulate incipient evolution of mammalian sex chromosomes
Source: Genome Biol. 2008 Jun 14;9(6):R98. doi: 10.1186/gb-2008-9-6-r98 (PMC2481430; doi:10.1186/gb-2008-9-6-r98)
Supplement: Additional data file 5 — Presented is a table showing the RNA editing degree of SNX22 neo-X allele in male black muntjacs is higher than that in female. [file gb-2008-9-6-r98-S5.doc]

**The RNA editing degree of *SNX22*** neo-X allele in male black muntjacs is higher than that in female.

|  | Edited form in male | Normal form in male | Edited form in female | Normal form in female |
| --- | --- | --- | --- | --- |
| Test 1 | 5 | 0 | 1 | 24 |
| Test 2 | 5 | 0 | 0 | 25 |

Note: RT-PCR products from both male and female black muntjac were subjected to TA-cloning and 25 positive clones were randomly picked for further sequencing. Experiments were done twice and results were shown in the table. In males, five neo-X transcripts detected are all edited, the other 20 are from neo-Y allele, but in female only one neo-X transcript is edited.
